# Augmented interpretation of HER2, ER, and PR in breast cancer by artificial intelligence analyzer: enhancing interobserver agreement through a reader study of 201 cases

**DOI:** 10.1186/s13058-024-01784-y

**Published:** 2024-02-23

**Authors:** Minsun Jung, Seung Geun Song, Soo Ick Cho, Sangwon Shin, Taebum Lee, Wonkyung Jung, Hajin Lee, Jiyoung Park, Sanghoon Song, Gahee Park, Heon Song, Seonwook Park, Jinhee Lee, Mingu Kang, Jongchan Park, Sergio Pereira, Donggeun Yoo, Keunhyung Chung, Siraj M. Ali, So-Woon Kim

**Affiliations:** 1 Department of Pathology, Yonsei University College of Medicine, Seoul, Republic of Korea; 2 Department of Pathology, Seoul National University College of Medicine, Seoul, Republic of Korea; 3 Lunit, Seoul, Republic of Korea; 4 Department of Pathology, Kyung Hee University Hospital, Kyung Hee University College of Medicine, Seoul, Republic of Korea

**Keywords:** Artificial intelligence (AI), Breast cancer, Concordance, Digital pathology, Estrogen receptor (ER), Human epidermal growth factor receptor 2 (HER2), Progesterone receptor (PR), Whole-slide image (WSI)

## Abstract

**Background:**

Accurate classification of breast cancer molecular subtypes is crucial in determining treatment strategies and predicting clinical outcomes. This classification largely depends on the assessment of human epidermal growth factor receptor 2 (HER2), estrogen receptor (ER), and progesterone receptor (PR) status. However, variability in interpretation among pathologists pose challenges to the accuracy of this classification. This study evaluates the role of artificial intelligence (AI) in enhancing the consistency of these evaluations.

**Methods:**

AI-powered HER2 and ER/PR analyzers, consisting of cell and tissue models, were developed using 1,259 HER2, 744 ER, and 466 PR-stained immunohistochemistry (IHC) whole-slide images of breast cancer. External validation cohort comprising HER2, ER, and PR IHCs of 201 breast cancer cases were analyzed with these AI-powered analyzers. Three board-certified pathologists independently assessed these cases without AI annotation. Then, cases with differing interpretations between pathologists and the AI analyzer were revisited with AI assistance, focusing on evaluating the influence of AI assistance on the concordance among pathologists during the revised evaluation compared to the initial assessment.

**Results:**

Reevaluation was required in 61 (30.3%), 42 (20.9%), and 80 (39.8%) of HER2, in 15 (7.5%), 17 (8.5%), and 11 (5.5%) of ER, and in 26 (12.9%), 24 (11.9%), and 28 (13.9%) of PR evaluations by the pathologists, respectively. Compared to initial interpretations, the assistance of AI led to a notable increase in the agreement among three pathologists on the status of HER2 (from 49.3 to 74.1%, *p* < 0.001), ER (from 93.0 to 96.5%, *p* = 0.096), and PR (from 84.6 to 91.5%, *p* = 0.006). This improvement was especially evident in cases of HER2 2+ and 1+, where the concordance significantly increased from 46.2 to 68.4% and from 26.5 to 70.7%, respectively. Consequently, a refinement in the classification of breast cancer molecular subtypes (from 58.2 to 78.6%, *p* < 0.001) was achieved with AI assistance.

**Conclusions:**

This study underscores the significant role of AI analyzers in improving pathologists' concordance in the classification of breast cancer molecular subtypes.

**Supplementary Information:**

The online version contains supplementary material available at 10.1186/s13058-024-01784-y.

## Background

Breast cancer has typically been classified into molecular intrinsic subtypes based on routine immunohistochemistry (IHC), including human epidermal growth factor receptor 2 (HER2), estrogen receptor (ER), and progesterone receptor (PR) [1]. The classification based on the presence of these receptors and expression level plays a significant role in both prognostic assessment and determining the most appropriate treatment approach [1, 2]. For example, the level of HER2 expression is crucial for predicting the therapeutic response to trastuzumab, the canonical HER2-targeted monoclonal antibody and the level of ER expression is essential for forecasting the treatment response to tamoxifen, a selective ER modulator [3, 4]. Recently, novel therapeutic agents such as trastuzumab deruxtecan, an antibody–drug conjugate (ADC) of trastuzumab and a ‘tecan,’ have emerged, demonstrating compelling results in particular for the treatment of HER2-low (HER2 1+ or HER2 2+ without in situ hybridization [ISH] amplification) breast cancer [5]. Consequently, this underscores the importance of a precise evaluation of the expression levels of the protein targets via IHC, as these results play a pivotal role in tailoring and optimizing therapeutic strategies.

However, significant interobserver and interlaboratory variations among pathologists have been noted in the evaluation of HER2, ER, and PR status, particularly for possible HER2-low cases [6–8]. Such inconsistencies might directly influence patient survival outcomes by affecting the selection of optimal treatment strategies [8, 9].

Recently, advancements and increasing deployment of digital pathology systems have paved the way for numerous strategies to analyze and quantify digital whole-slide images (WSIs) [10, 11]. Specifically, this permits the application of AI algorithms designed for various fields of oncology, including image analysis [12]. In early studies, such algorithms have shown significant potential to reduce interobserver variability among pathologists which has correlated with the improved prediction of response to treatment [13–17].

There are several approaches to utilize AI algorithms to assess HER2, ER, and PR status in breast cancer. Previous studies demonstrated the improved consistency and accuracy in the evaluation of HER2 status by pathologists with AI assistance, including HER2-low cases [18–21]. AI algorithms have also demonstrated excellent agreement with pathologists' interpretation of ER and PR status [22, 23]. Although these studies have reported on the development of individual AI models for the assessment of HER2 or ER/PR, few studies have evaluated the impact of AI-aided analysis on assessing HER2, ER, and PR comprehensively for a single patient cohort.

In this study, we conducted a comprehensive AI-assisted reader study to evaluate HER2, ER, and PR status on the same patients. Our aim was to examine whether AI assistance could ameliorate the interobserver variability associated with the evaluation of HER2, ER, and PR status in breast cancer, and subsequent impact on the determination of molecular subtypes of breast cancer.

## Methods

### Dataset for HER2 and ER/PR analyzer development

An AI-powered HER2 analyzer of breast, Lunit SCOPE HER2 (Lunit, Seoul, Republic of Korea) was developed with 1259 HER2 IHC-stained WSIs of breast cancer, consisting of 880, 253, and 126 for training, tuning, and internal test, in each set. An AI-powered ER/PR analyzer of breast, Lunit SCOPE ER/PR was developed with 1210 ER/PR IHC-stained WSIs of breast cancer, consisting of 782, 287, and 141 WSIs for training, tuning, and internal test, in each set. Further information on the dataset is described in Additional file 1: Supplementary Methods.

### Data preprocessing for model development

Patches of a predefined area (0.04 mm^2^ for cell and 2.54 mm^2^ for tissue) were extracted from WSIs. Those images had a normalized micron per pixel resolution of 0.19 μm (1024 * 1024 pixels for cell and 8192 * 8192 pixels for tissue) and were used as input for the AI model development. In the patch level, we prevented dataset leakage by following the classification of training, tuning, and internal tests classified by WSIs. Additional file 1: Table S1 shows the numbers of WSIs and patches assigned to training, tuning, and internal test sets. All patches were annotated by board-certified pathologists, and further information about annotation results is mentioned in Additional file 1: Supplementary Methods, Additional file 1: Tables S2 and S3.

### Development of AI model—Cell Detection Model

The cell detection models of both HER2 and ER/PR identify all tumor cells. For HER2, the model also identifies other cells (OTs or non-tumor cells). Beyond detecting the location of cells, the model is also proficient in identifying varying intensity levels associated with each cell. Therefore, the HER2 model can detect a total of five cell classes, including four tumor cells (3+, 2+, 1+, and 0), and OTs, while ER/PR model can detect four cell classes of tumor cells (3+, 2+, 1+, and 0). This cell detection task was approached as a dense segmentation challenge, utilizing the DeepLabv3+ segmentation model with a ResNet-34 for feature extraction [24, 25]. Additional details of the AI model are described in Additional file 1: Supplementary Methods.

### Development of AI model—Tissue segmentation model

The tissue segmentation model evaluates each pixel of the input to determine if it belongs to specific classes. The HER2 model can segment CA (cancer area; invasive breast cancer), CIS (carcinoma in situ), or BG (background; any tissue area that does not belong to CA or CIS), while the ER/PR model can segment CA or BG (including CIS). The model employs a DeepLabv3, complemented with a ResNet-101 for feature extraction. Additional details of the AI model are described in Additional file 1: Supplementary Methods.

### Scoring algorithms of HER2 and ER/PR analyzer

The HER2 analyzer and ER/PR analyzer evaluated the expression of HER2 and ER/PR at the slide level by merging the results from the cell detection model and the tissue segmentation model from a WSI. The HER2 analyzer counted the HER2-positive tumor cells in the CA area and calculated the proportion of each tumor cell class (3+, 2+, 1+, and 0). The ER/PR analyzer also counted the ER/PR-positive tumor cells in the CA area, but calculated the proportion of any positive tumor cells (regardless of intensity level). The slide-level expression level of HER2, ER, and PR was categorized by the American Society of Clinical Oncology (ASCO)/College of American Pathologists (CAP) guidelines [26, 27].

### External test dataset and reader study

An external test set was selected from Kyung Hee University Hospital (Seoul, Republic of Korea). The inclusion criteria for this study were cases diagnosed as breast cancer by pathologists between January 2018 and December 2021 and had all of the matched HER2, ER, and PR slides. All slides were stained with Ventana anti-HER2/neu (4B5) (Ventana Medical Systems, Tucson, AZ, USA) for HER2, Novocastra Liquid Mouse Monoclonal Antibody Estrogen Receptor (NCL-L-ER-6F11) (Novocastra Laboratories, Newcastle, UK) for ER, and Novocastra Liquid Mouse Monoclonal Antibody Progesterone Receptor (PGR-312-L-CE, PGR-312-L-CE-S) for PR. All slides were scanned with a P1000 scanner with 40× magnification and were inferred by the AI algorithms mentioned above.

Three board-certified pathologists (M.J., S.-W.K., and S.G.S.), each from different hospitals, independently evaluated the scanned HER2, ER, and PR IHC slides in accordance with the guidelines using a digital visualizer [26, 27]. They could zoom in or out of each scanned WSI through the digital visualizer to determine the expression level of HER2/ER/PR at the slide level (Additional file 1: Figure S1). They annotated WSI-level HER2 expression level as 3+ (positive), 2+ (equivocal), 1+ (negative but low), and 0 (negative), and ER/PR expression level as positive (> 10% of cell staining), low positive (1–10% of cell staining), and negative (< 1% of cell staining) without AI inference results. Per ASCO/CAP guidelines updated in 2020, PR cases were categorized as negative and positive, but in this study, low positives were additionally categorized separately to make comparable results with ER cases.

**Fig. 1:**
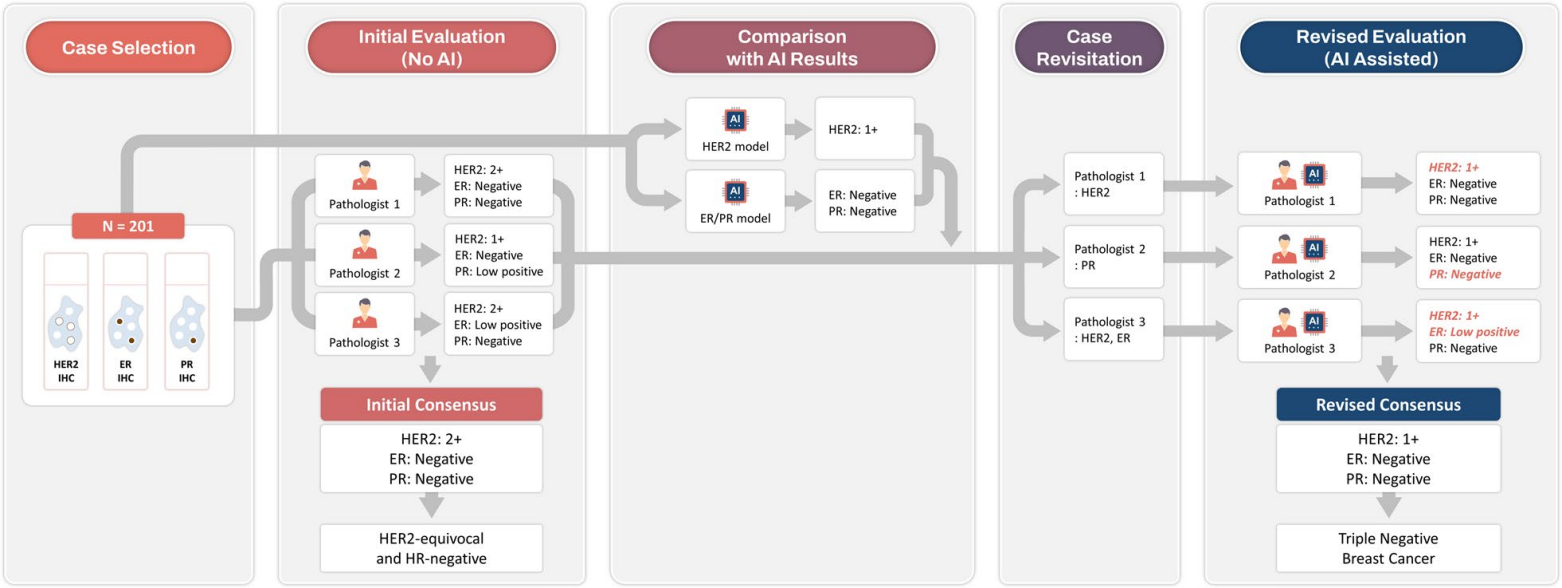
A schematic flow of the reader study (AI: artificial intelligence, ER: estrogen receptor, HER2: human epidermal growth factor receptor 2, PR: progesterone receptor)

If a pathologist's evaluation (any of HER2, ER, and PR IHC) did not match the result from AI analyzers, each pathologist revisited the case using AI's inferred results and reevaluated it independently. In this phase, the pathologists used the same digital visualizer as before, with the AI inference results added. The visualizer showed the total numbers of IHC-positive tumor cells (including their intensity) and negative tumor cells, the expression level of HER2/ER/PR, and the coordinate of each cell on the WSIs and segmentation of tissue (Additional file 1: Figure S1). Figure 1 displays the flow of the reader study.

Consensus was determined by combining the results of independent evaluations from three pathologists. If all three pathologists agreed to the results, the case was categorized as concordant; if only two agreed, it was categorized as partially concordant, and otherwise as discordant. A consensus result is defined as an evaluation result that at least two out of three people agree on. Therefore, a concordant or partially concordant case can have a consensus result. If a case was altogether discordant, those cases were labeled as no consensus. The degree of agreement between pathologists is measured in both initial (without AI assistance) and revised (revisiting and reevaluating with AI assistance in case of disagreement with AI results) results.

This study was approved by the Institutional Review Board (IRB) of Kyung Hee University Hospital (IRB no. KHUH 2022-01-035). Informed consent was waived by the IRB because of the retrospective design of the study and the anonymized clinical data used in the analysis. The study was performed in accordance with the Declaration of Helsinki.

### Statistical analysis

F1 score and intersection over union (IoU) were applied to evaluate the performance of the cell model and the tissue model, respectively. Agreement rate or quadratic weighted kappa value between raters or AI analyzers was evaluated as overall agreement. Categorical variables were compared using the Chi-square test/Fisher’s exact test or McNemar test. All statistical analyses were performed using Python 3.7 and R version 4.0.3 software (R Foundation for Statistical Computing, Vienna, Austria).

## Results

### Performance of cell detection and tissue segmentation models—HER2 and ER/PR

In the HER2 model, the cell detection showed the best performance for OT, but had the lowest performance for 2+ tumor cells. For the ER/PR model, the highest cell detection performance was observed in 3+ tumor cells, while the performance was lowest in 1+ tumor cells. The tissue segmentation model in the HER2 model achieved better performance on CA than on CIS, while those in the ER/PR model achieved performances of CA comparable to the HER2 model. Further detail of cell detection and tissue segmentation model performance is described in Additional file 1: Supplementary Results and Additional file 1: Tables S4–S7.

### Clinical information of the reader study set

We retrospectively collected data and found a total of 201 breast cancer patients with all three IHC types of HER2, ER, and PR slides. Of these, 199 (99.0%) were female and 2 (1.0%) were male. The median age at the time of specimen collection was 57 years, with ages ranging from a minimum of 28 to a maximum of 84 years. The majority of cases were surgical excision specimens (*N* = 189, 94.0%), and American Joint Committee on Cancer (AJCC) stage 1A cases were the most common (*N* = 87, 43.3%). Table 1 summarizes the clinical information of the cases.

**Table 1:** Clinical information of external test dataset (*N* = 201)

Type	Category	Number of cases (%)
Sex	Female	199 (99.0%)
	Male	2 (1.0%)
Age group	≤ 40	14 (7.0%)
	41–50	55 (27.4%)
	51–60	50 (24.9%)
	61–70	49 (24.4%)
	≥ 71	33 (16.4%)
Specimen	Biopsy	12 (6.0%)
	Surgical excision	189 (94.0%)
Stage	1A	90 (44.8%)
	1B	1 (0.5%)
	2A	56 (27.9%)
	2B	23 (11.4%)
	3A	16 (8.0%)
	3B	1 (0.5%)
	3C	8 (4.0%)
	4	6 (3.0%)

### WSI-level assessment of HER2/ER/PR by pathologists in the reader study set

**Fig. 2:**
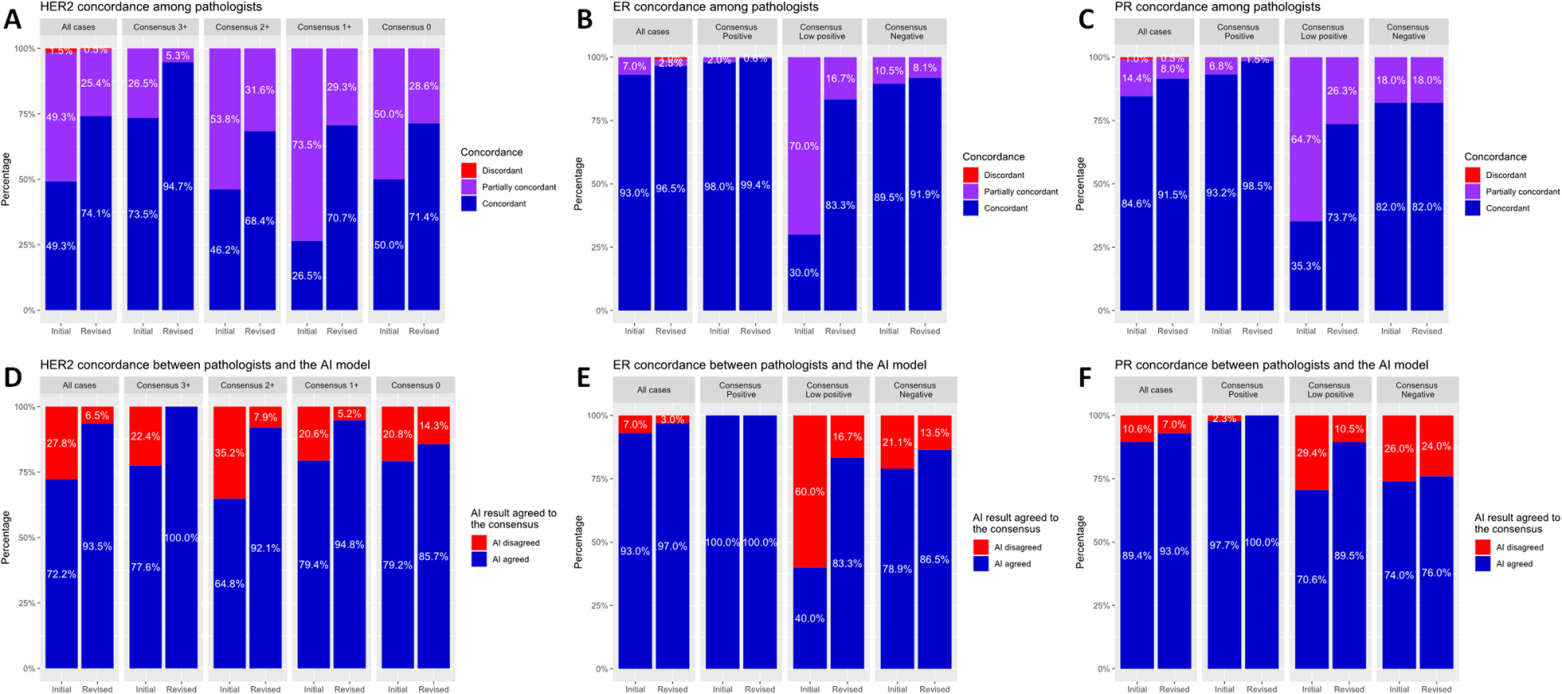
Concordance among pathologists in HER2 (human epidermal growth factor receptor 2) dataset (**A**), ER (estrogen receptor) dataset (**B**), and PR (progesterone receptor) dataset (**C**). Concordance between the consensus of pathologists and the AI (artificial intelligence) analyzer in HER2 dataset (**D**), ER dataset (**E**), and PR dataset (**F**)

For the HER2 WSI, there were 99 (49.3%) concordant cases, 99 (49.3%) partially concordant cases, and three (1.5%) discordant cases (Fig. 2A). In the consensus results of the pathologists, there were 49 (24.4%) cases of HER2 3+, 91 (45.3%) cases of HER2 2+, 34 (16.9%) cases of HER2 1+, 24 (11.9%) cases of HER2 0, and 3 (1.5%) cases with no consensus. Consensus-classified HER2 3+ had the highest concordance rate (73.5%) and HER2 1+ had the lowest (26.5%). Agreement rates and quadratic weighted kappa values between the two pathologists were 66.2%/0.803 (pathologist 1 [P1] and pathologist 2 [P2]), 77.6%/0.843 (P1 and pathologist 3 [P3]), and 53.2%/0.709 (P2 and P3), respectively (Additional file 1: Figure S2A–C).

For the ER WSI, there were 187 (93.0%) concordant cases and 14 (7.0%) partially concordant cases (Fig. 2B). In the pathologists’ consensus results, there were 153 (76.1%) cases of ER-positive, 10 (5.0%) cases of ER-low positive, and 38 (18.9%) cases of ER-negative. Consensus-classified ER-positive had the highest concordance rate (98.0%) and ER-low positive had the lowest (30.0%). Agreement rates and quadratic weighted kappa values between the two pathologists were 95.5%/0.940 (P1 and P2), 95.5%/0.952 (P1 and P3), and 95.0%/0.949 (P2 and P3), respectively (Additional file 1: Figure S3A–C).

On the PR slides, there were 170 (84.6%) concordant cases, 29 (14.4%) partially concordant cases, and two (1.0%) discordant cases (Fig. 2C). In the pathologists’ consensus results, PR-positive, PR-low positive, PR-negative, and no consensus were 132 (65.7%) cases, 17 (8.5%) cases, 50 (24.9%) cases, and 2 (1.0%) cases, in each. Consensus-classified PR-positive had the highest concordance rate (93.2%) and PR-low positive had the lowest (35.3%). Agreement rates and quadratic weighted kappa values between the two pathologists were 90.0%/0.922 (P1 and P2), 90.5%/0.937 (P1 and P3), and 87.6%/0.906 (P2 and P3), respectively (Additional file 1: Figure S4A–C).

Overall, the pathologists' concordances for HER2/ER/PR IHC results are generally lower for the cases within IHC low positive category (i.e., HER2 2+ or 1+, ER-low positive, PR-low positive).

### Standalone performance of the AI analyzer

The AI analyzer had an agreement rate of 72.2% (143 out of 198, excluding three cases of no consensus) and quadratic weighted kappa value of 0.844 compared to the pathologists’ HER2 consensus result (Fig. 2D). Compared to pathologists, the AI analyzer tended to classify lower HER2 grades than the pathologists’ consensus (Additional file 1: Figure S5A). In the 99 cases where the three pathologists agreed, the AI results had an 89.9% (*N* = 89) agreement rate and quadratic weighted kappa value of 0.948 with the consensus result.

In the ER dataset, the AI analyzer had an agreement rate of 93.0% (*N* = 187) and quadratic weighted kappa value of 0.916 compared to the pathologists’ ER consensus result (Fig. 2E). In contrast to the HER2 AI analyzer, compared to pathologists, the AI analyzer tended to classify higher ER grades than the pathologists’ consensus (Additional file 1: Figure S5B). In the 187 cases where the three pathologists agreed, the AI results had a 96.3% (*N* = 180) agreement rate and quadratic weighted kappa value of 0.938 with the consensus result.

In the PR dataset, the AI analyzer had an agreement rate of 89.4% (178 out of 199, excluding two cases of no consensus) and quadratic weighted kappa value of 0.902 compared to the pathologists’ PR consensus result (Fig. 2F). Similar to the ER AI analyzer, compared to pathologists, the AI analyzer tended to classify higher PR grades than the pathologists’ consensus (Additional file 1: Figure S5C). In the 170 cases where the three pathologists agreed, the AI results had a 93.5% (*N* = 159) agreement rate and quadratic weighted kappa value of 0.941 with the consensus result.

### Change in HER2/ER/PR interpretation after AI assistance

**Fig. 3:**
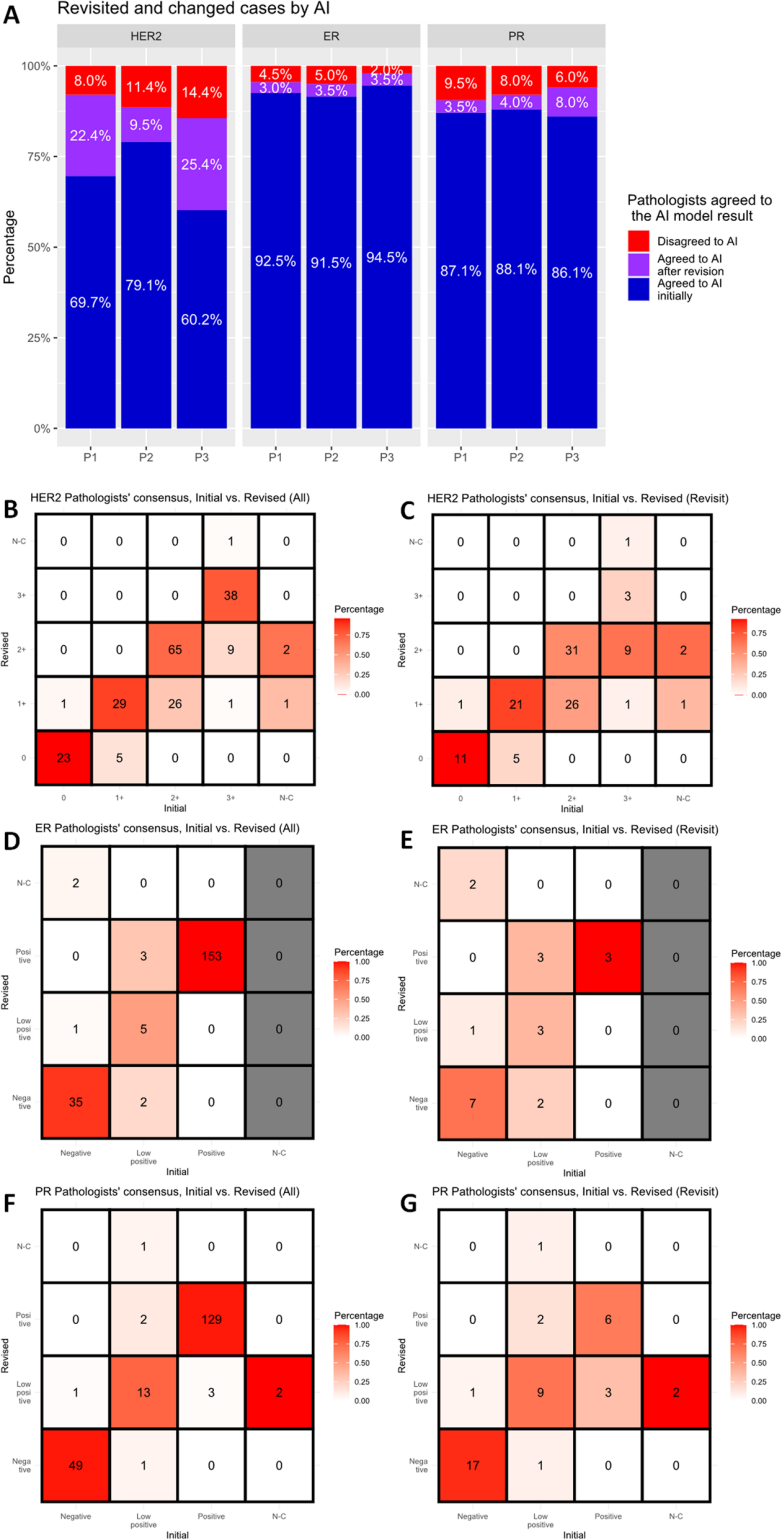
**A** Proportion of revisited and revised cases by artificial intelligence (AI) analyzer in Pathologist 1 (P1), Pathologist 2 (P2), and Pathologist 3 (P3). Initial and revised pathologists’ consensus of HER2 (human epidermal growth factor receptor 2) in All cases (**B**) or revisited cases only (**C**). Initial and revised pathologists’ consensus of ER (estrogen receptor) in All cases (**D**) or revisited cases only (**E**). Initial and revised pathologists’ consensus of PR (progesterone receptor) in All cases (**F**) or revisited cases only (**G**)

The cases that were discordant between the pathologists and AI were reevaluated by the pathologists. In the HER2 dataset, the numbers of cases revisited were as follows: 61 cases (30.3%) for P1, 42 cases (20.9%) for P2, and 80 cases (39.8%) for P3 (Fig. 3A). Among these, the HER2 classification was revised by the pathologists in 46 cases (75.4%) for P1, 19 cases (45.2%) for P2, and 54 cases (67.5%) for P3. After revisiting, pathologists’ consensus results were changed; HER2 3+, HER2 2+, HER2 1+, HER2 0, and no consensus were 38 (18.9%) cases, 76 (37.8%) cases, 58 (28.9%) cases, 28 (13.9%) cases, and 1 (0.5%) cases, in each (Fig. 3B). In cases (*N* = 112) of revisit, HER2 results tended to change to a lower grade (e.g., HER2 1+ to 0, 2+ to 1 +) (Fig. 3C, Additional file 1: Figures S6A-C). After revisiting, the number of cases in which all three pathologists agreed increased significantly from 99 (49.3%) to 149 (74.1%) cases (*p* < 0.001) (Fig. 2A). Compared to the initial evaluation, the revised evaluation improved concordance at all HER2 expression levels, but especially at HER2 2+ and 1+, with a significant increase in concordance from 46.2 to 68.4% and 26.5 to 70.7%, respectively (Fig. 2A). Agreement rates and quadratic weighted kappa values between the two pathologists after revision were changed to 84.6%/0.914 (P1 and P2), 84.6%/0.911 (P1 and P3), and 78.6%/0.875 (P2 and P3), respectively (Additional file 1: Figure S2D–F).

In ER dataset, there were 15 (7.5%) cases for P1, 17 (8.5%) cases for P2, and 11 (5.5%) cases for P3 that went to revisit (Fig. 3A). Among them, pathologists revised ER classification in 6 (40.0%), 8 (47.1%), and 7 (63.6%) cases, in each. After revisiting, pathologists’ consensus results were changed; ER-positive, ER-low positive, ER-negative, and no consensus were 156 (77.6%) cases, 6 (3.0%) cases, 37 (18.4%) cases, and 2 (1.0%) cases, in each (Fig. 3D). In cases (*N* = 21) of revisit, ER results tended to change to a higher grade (e.g., ER-negative to weak positive, weak positive to positive) (Fig. 3E, Additional file 1: Figures S6D–F). After revisiting, the number of cases in which all three pathologists agreed increased from 187 (93.0%) to 194 (96.5%) cases, but this increase was not statistically significant (*p* = 0.096) (Fig. 2B). Compared to the initial pathologist interpretation, the revised evaluation improved concordance among the pathologists at all ER expression levels, but especially at ER-low positive, with a significant increase in concordance from 30.0 to 83.3% (Fig. 2B), which concurred with the acceptance of the AI’s inference of ER-low positivity (Fig. 2E). Agreement rates and quadratic weighted kappa values between the two pathologists after revision were changed to 97.5%/0.967 (P1 and P2), 97.5%/0.967 (P1 and P3), and 97.1%/0.975 (P2 and P3), respectively (Additional file 1: Figure S3D–F).

In PR dataset, there were 26 (12.9%) cases for P1, 24 (11.9%) cases for P2, and 28 (13.9%) cases for P3 that went to revisit (Fig. 3A). Among them, pathologists revised PR classification in 7 (26.9%), 9 (37.5%), and 16 (57.1%) cases, in each. After revisiting, pathologists’ consensus results were changed; PR-positive, PR-low positive, PR-negative, and no consensus were 131 (65.2%) cases, 19 (9.5%) cases, 50 (24.9%) cases, and 1 (0.5%) cases, in each (Fig. 3F). In cases (*N* = 42) of revisit, changes in PR results did not skew toward higher or lower (Fig. 3G, Additional file 1: Figures S6G–I). After revisiting, the number of cases in which all three pathologists agreed increased significantly from 170 (84.6%) to 184 (91.5%) cases (*p* = 0.006) (Fig. 2C). Compared to the initial evaluation, the revised evaluation improved concordance among the pathologists especially at PR-low positive, with a significant increase in concordance from 35.3 to 73.7% (Fig. 2C). The pathologist–AI concordance also increased in PR-low positive cases, from 70.6 to 89.5% (Fig. 2F). Agreement rates and quadratic weighted kappa values between the two pathologists after revision were changed to 94.5%/0.962 (P1 and P2), 94.5%/0.953 (P1 and P3), and 93.5%/0.955 (P2 and P3), respectively (Additional file 1: Figure S4D–F).

### Molecular Subtypes of breast cancer after AI assistance

**Fig. 4:**
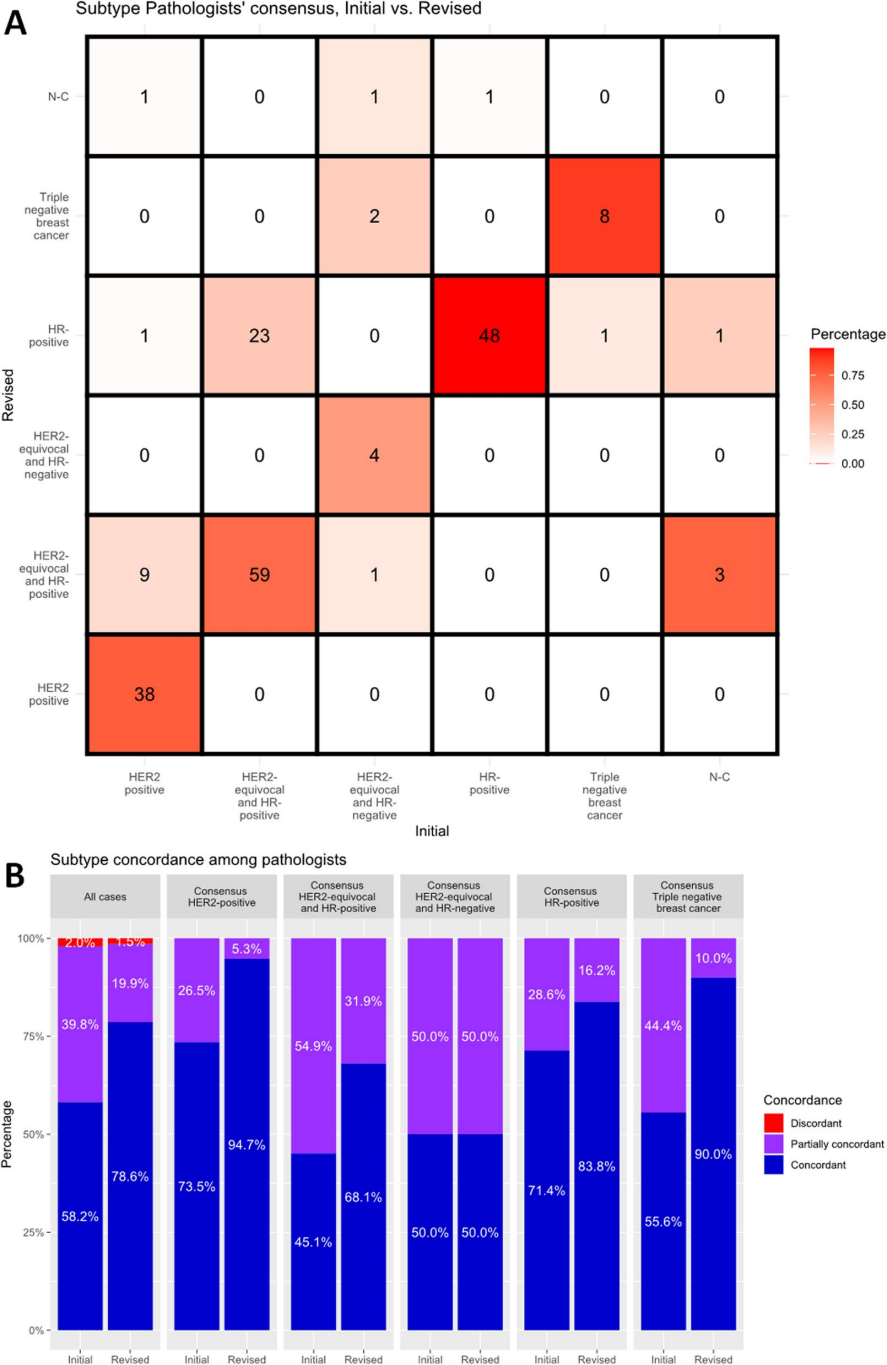
**A** Initial and revised pathologists’ consensus of subtype. **B** Initial and revised concordance rates of subtype among pathologists (ER: estrogen receptor, HER2: human epidermal growth factor receptor 2, PR: progesterone receptor)

In this study, breast cancer molecular subtypes can be divided into the following: (1) HER2-positive: HER2 3+; (2) HER2-equivocal and HR-positive: HER2 2+ with at least one ER/PR-positive (including low positive); (3) HER2-equivocal and HR-negative: HER2 2+ with both ER/PR-negative; (4) HR-positive: HER2 1+ or 0 with at least one ER/PR-positive (including low positive); (5) triple-negative breast cancer (TNBC): HER2 1+ or 0 with both ER/PR-negative. HER2-equivocal and HR-positive subtype had the highest number of cases at initial evaluation (*N* = 82, 40.8%), followed by HER2-positive subtype and HR-positive subtype (*N* = 49, 24.4% in each), TNBC subtype (*N* = 9, 4.5%), HER2-equivocal and HR-negative subtype (*N* = 8, 4.0%), and no consensus (*N* = 4, 2.0%), respectively (Fig. 4A). The number of cases where subtypes were agreed between all pathologists was 117 (58.2%) cases. Of these, the initial concordance rates of pathologist interpretation were lowest for HER2-equivocal and HR-positive subtype at 45.1% and highest for HER2-positive at 73.5% (Fig. 4B).

After revisiting by AI analyzers, HR-positive subtype was the most common with 74 (36.8%) cases, followed by HER2-equivocal and HR-positive subtype with 72 (35.8%) cases, HER2-positive subtype with 38 (18.9%) cases, TNBC subtype with 10 (5.0%) cases, HER2-equivocal and HR-negative subtype with 4 (2.0%) cases, and no consensus with 3 (1.5%) cases (Fig. 4A). Of note, 18.4% (9/49) of HER2-positive cases were reclassified as a HER2-equivocal and HR-positive type, and 28.0% (23/82) of the HER2-equivocal and HR-positive cases were reclassified as an HR-positive type. The number of cases where subtypes were agreed between all pathologists significantly increased from 117 to 158 cases (58.2 to 78.6%, *p* < 0.001). The concordance rate increased for all subtypes except for the HER2-equivocal and HR-negative subtype (Fig. 4B).

### Analyze factors that can affect pathologists’ concordance or AI analyzer performance

**Fig. 5:**
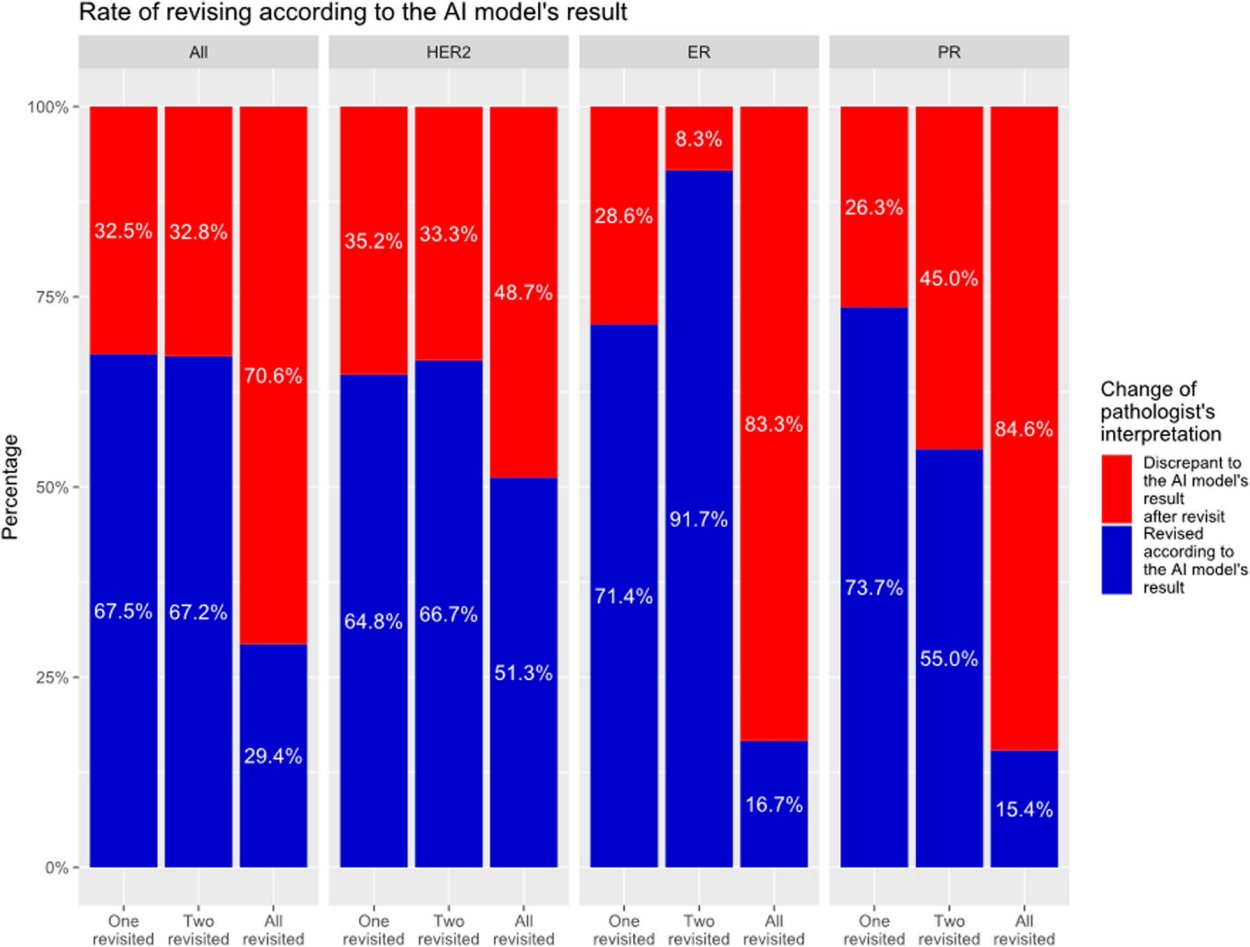
Rate of revising by pathologists according to the artificial intelligence (AI) analyzer’s results, when one, two, or all three pathologists revisited (ER: estrogen receptor, HER2: human epidermal growth factor receptor 2, PR: progesterone receptor)

We further analyzed the interaction between the pathologist and the AI analyzer. First, a total of 304 revisiting requests were made to the three pathologists, of which 166 (54.6%) revised their interpretation according to the AI analyzer’s results. HER2 was most likely to be changed on revisit (62.8%) and PR was least likely (39.7%) (Fig. 5). Depending on the number of pathologists who requested to revisit, 67.5% (54/80) of cases where only one pathologist was asked to revisit the case were revised based on the AI analyzer results, but only 29.4% (30/102) of cases where all three pathologists were asked to revisit the case were revised.

Next, we defined a complete failure of the AI analyzer as a case where all three pathologists were requested to revisit and none of them changed their initial interpretations. This occurred in a total of 10 cases. Within these, two cases exhibited specific failures in HER2, without affecting ER/PR evaluations. Four cases demonstrated failures solely in PR, with no impact on HER2 and ER evaluations. The remaining four cases presented concurrent failures in both ER and PR, but not in HER2.

**Fig. 6:**
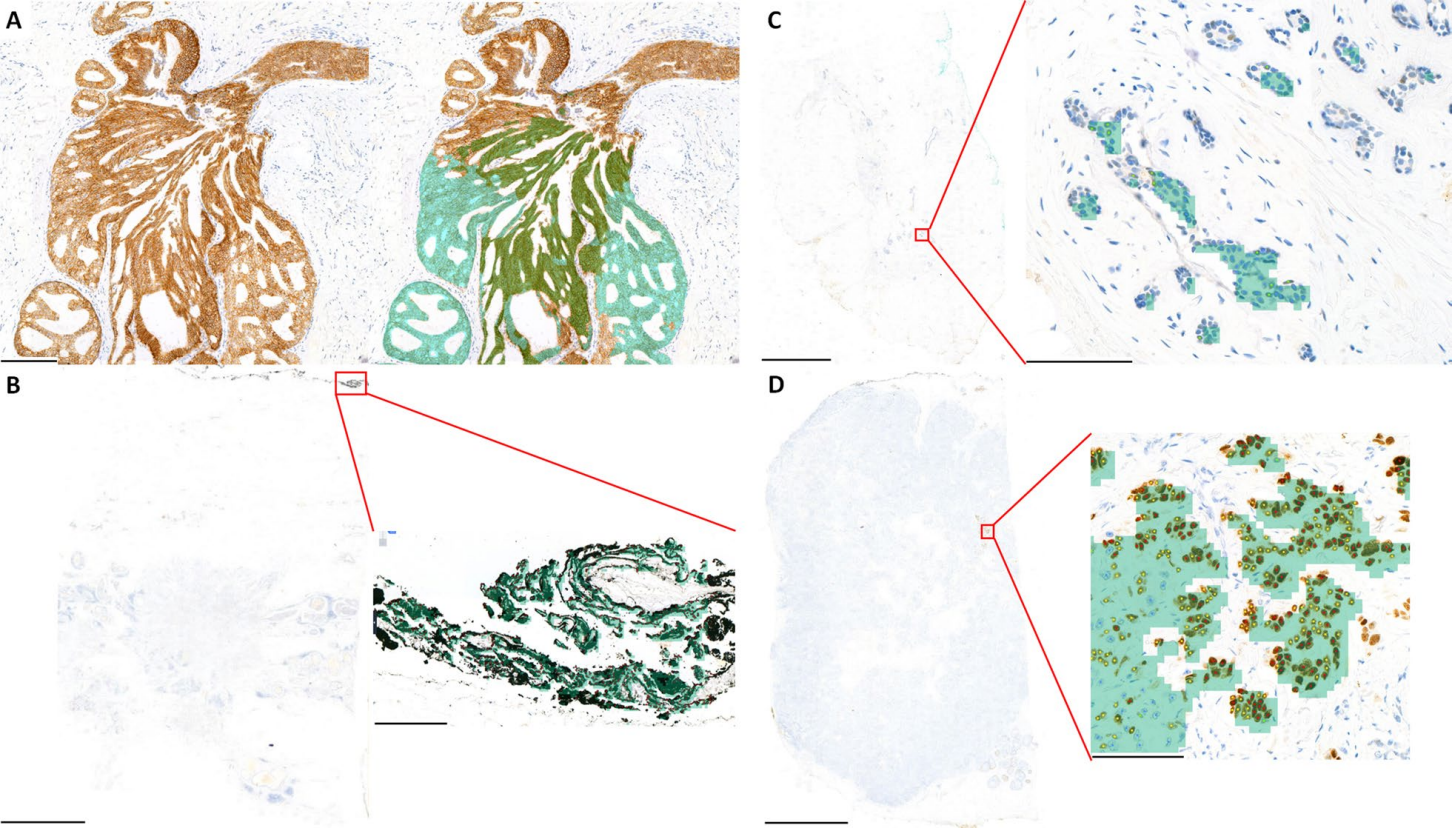
**A** Carcinoma in situ areas containing HER2 (human epidermal growth factor receptor 2)-stained cells were classified as cancer area (CA) by HER2 analyzer (bar: 200 μm). **B** ER (estrogen receptor)/PR (progesterone receptor) analyzer recognized the inked area at the margin of the tissue as a CA with ER-positive tumor cells (bar left: 5 mm, right: 200 μm). **C** ER/PR analyzer barely caught the CA through the entire area of PR-stained slide (bar left: 5 mm, right: 100 μm). **D** In a PR-stained slide, pathologists focused the visible clusters of positive tumor cells at low magnification and classified them as low positive all together even after revision. In contrast, ER/PR analyzer counted all the tumor cells and interpreted them as negative (bar left: 5 mm, right: 100 μm)

In the case of HER2, the AI analyzer classified cases that were pathologists’ consensus 1+ in both cases as 2+. In those cases, normal ductal or CIS areas containing HER2-stained cells were classified as CA (Fig. 6A). For the 4 cases with ER/PR overlapped, the pathologists’ consensus was negative, but the AI analyzer classified them as positive or low positive. These were due to the AI analyzer recognizing the inked area at the margin of the tissue as a CA with positive tumor cells (Fig. 6B). Of the four cases with no ER issues and only PR issues, two were caused by ink, as above. In one case where the tumor cells showed mild atypia and a low density of tumor cell clusters, the AI analyzer made a poor interpretation, barely catching the CA throughout the entire area of a slide (Fig. 6C). In the last case, the three pathologists concordantly classified it as low positive even after revision because there were clearly visible clusters of positive tumor cells at low magnification on the slide. However, the AI analyzer counted all the tumor cells and read them as negative (Fig. 6D).

## Discussion

In this study, we found that when pathologists assessed the expression of HER2, ER, and PR in breast cancer with the assistance of an AI algorithm, the concordance of their individual readings increased.

The ASCO/CAP guidelines strongly recommend HER2, ER, and PR testing of invasive breast cancers [26, 27]. The objective of these guidelines is to enhance the accuracy of these diagnostic assays, which enable clinicians to identify breast cancer patients who will benefit most from endocrine therapy or HER2-targeted therapy. Recent advancements, notably the emergence of HER2-targeted ADC, have demonstrated improved survival outcomes in breast cancer patients with HER2 IHC scores of 1+ or 2+ (HER2-low when combined with knowledge of negative *ERBB2* amplification status). This underscores the increasing necessity for a precise interpretation of HER2 testing [5].

In the evaluation of HER2 status via IHC, pronounced interobserver discrepancies among pathologists have been identified. While there is a high consensus among pathologists for 0 and +3 staining, agreement levels drop significantly for 1+ and 2+ staining [6]. Consistent with this observation, another study found a 26% agreement between HER2 IHC scores of 0 and 1+ [7]. As for ER and PR evaluations using IHC, although classifications related to the ER and PR status of tumors generally demonstrate good to excellent agreement, considerable variations both within and between laboratories have been reported [8, 28]. Preceding these laboratory and observer variations, preanalytic factors can also affect the determination of IHC interpretations such as anatomic origin of the tissue, storage conditions, and fixation methods. Such discrepancies can lead to variations in the determination of IHC status, and thus subtyping, treatment and clinical outcomes [9, 29].

Digital pathology has been rapidly spreading, overcoming issues related to image capacity and lack of standardization. [30, 31]. With the spreading of digital pathology, there have been several attempts to introduce AI algorithms in the field of pathology to enhance standardization and compensate for analytic variability [10, 11]. Some studies have evidenced that these algorithms not only diminish interobserver variability but can also prognosticate the treatment response to immunotherapy by assessing programmed death ligand 1 (PD-L1) expression or tumor infiltrating lymphocytes [13, 14, 16, 17].

Previous computational assessments, whether rule-based or utilizing machine learning models, have shown promise in evaluating the IHC status of HER2, ER, and PR, typically reporting a high level of concordance between pathologists’ manual scoring and computational assessment [32–36]. However, most of those algorithms do not address tasks with parameters beyond their specific training set due to a lack of robustness and importantly may require human intervention to extract features, making these less suitable for the scale of clinical workflow. Furthermore, many such early models are circumscribed to analyzing tissue microarray images or require the selection of specific regions of interest (ROI) for analysis instead of WSIs [33–35].

The advent of large-scale datasets, coupled with the evolution of AI algorithms and drastically increasing computing power, have paved the way for deep learning (DL)-based AI models which mimic algorithmic structures of the human brain that exhibit impressive alignment with pathologists' assessments [18–23]. An AI model has shown a potential to be included in clinical digital workflow and another model has shown robustness across variable environmental factors such as staining systems or types of scanners [22, 23]. However, a common thread of limitation in most studies is their focus on individual AI analyzers assessing HER2, ER, or PR. Even in studies where AI was used for multiple biomarkers, concurrent evaluations on the same patient remained rare [23, 37].

The Al analyzer developed in this study was based on the DL algorithm and encompasses models for HER2 and ER/PR which each also contains both a cell detection model and a tissue segmentation model. In a previous report, an AI-powered PD-L1 analyzer based on a cell model alone occasionally misidentified normal epithelial cells as PD-L1 negative tumor cells [13]. In this study, by combining the results from cell and tissue models, some falsely detected tumor cells in areas outside ROI could be excluded.

In the context of the cell detection model, many discrepancies were observed, mainly in cell classes where the AI analyzer’s assessment differed by a single intensity grade, such as changing from a 2+ tumor cell to a 1+ tumor cell. In contrast, cases where the AI analyzer misjudged the intensity by more than two grades, like from a 3+ tumor cell to a negative (0) tumor cell, were less common. Another study also suggested that AI models might have reduced accuracy for mid-range grades compared to extreme grades [38].

In our study, the ER/PR analyzer exhibited relatively high agreement with the pathologist's interpretation because it focused solely on the proportion of positive or negative tumor cells, without considering intensity. This meant that even if the cell model misidentified a 2+ tumor cell as a 1+ tumor cell, it did not significantly affect the final result. However, the HER2 analyzer had lower agreement with the pathologist's interpretation because it needed to consider both intensity and proportion. Still, as shown in Additional file 1: Tables S4 and S6, there were cases where 2+ tumor cells were misclassified as 1+ tumor cells and vice versa. However, since there are typically thousands or more tumor cells in a WSI, unless misclassifications are skewed toward a particular class, the overall impact of misdetection is somewhat mitigated. As a result, the AI analyzers developed in our study demonstrated reliable performance, even when tested on external datasets, as indicated by the high agreement between the AI analyzers and pathologists for HER2/ER/PR grades.

After revising with AI analyzer, concordance in the individual interpretation of each IHC type and for molecular subtypes derived from the combined results of IHC was enhanced. Importantly, these results showed a significant increase in concordance for the classes corresponding to the low-HER2 classification (HER2 IHC 1+ or 2+) and remarkable decrease in HER2-positive class by the AI assistance. Following AI-assisted revisit, pathologists reached a consensus to downgrade the 26 out of 91 (28.6%) initial 2+ cases to 1+ (Fig. 3B). This downgrading avoids (unnecessary) ISH testing for ERBB2 amplification as indicated for HER2 2+ cases as this would identify HER2-low cases, either 2+ and ISH−, or 1+. This carries immediate implications given the eligibility of HER2-low patients for Enhertu, the HER2 ADC [5].

When utilizing the AI analyzer as a tool for second opinions, as done in this study, pathologists can maintain their original workflow, requiring reinterpretation in only a select subset of cases (approximately 30% for HER2 and 10% for ER and PR). As a result, with AI assistance, pathologists can achieve a more precise interpretation. When prompted by the AI analyzer to reevaluate in this study, if one or two pathologists revised their initial judgments, corrections were made in two-thirds or more of the original interpretations. This implies the AI analyzer's capacity to aid pathologists as a ‘second-reader’ in harmonizing judgments that may diverge due to over- or underestimations [39]. However, when all three pathologists were requested to reevaluate by the AI analyzer in 102 cases, the correction rate of their initial assessments decreased to less than one-third (29.4%), compared to the correction rate of 67.5% (54 out of 80 cases) when only one pathologist was revisited. This may indicate situations with inaccurate tissue segmentation (e.g., misclassifying CIS as CA or mistaking a normal duct for CA). Given that this AI analyzer was developed from a training set with specimens consisting predominantly of CA, this issue can be addressed by expanding its training to include samples from other tissue types, especially CIS, which is in progress.

This study has several limitations. First, even with the collection of consecutive cases over years from a university hospital, the potential for selection bias must be acknowledged. Second, the scope of validation of the AI analyzer was primarily confined to enhancing the concordance of pathologist interpretation. A clinical validation, such as gauging the AI analyzer's impact on patients' survival outcomes, has yet to be performed. Third, in actual clinical practice, HER2-equivocal (2+) has a final evaluation of HER2 expression by the fluorescence in situ hybridization (FISH) test, which was not available in this study due to the limited number of cases with test results. Lastly, the external validation of the analyzer relied on a dataset from a single institution and was reviewed by a limited number of pathologists. To ensure effective integration of an AI analyzer in clinical practice, comprehensive validation is essential. This should include testing with diverse external cohorts and conducting ring studies that involve a larger number of cases and pathologists [40].

Despite the aforementioned limitations, our study has several strengths. First and foremost, the statuses of HER2, ER, and PR were concurrently evaluated within a consecutive cohort of cases. Moreover, concordance arose from the interpretations of multiple pathologists from several institutions, which emulates real-world practice. Notably, the AI analyzer for ER and PR was effective even when the antibodies used differed between model training and validation phases which supports the robustness of the analyzer as it can generalize across different antibodies.

## Conclusions

In conclusion, we reported that pathologists' use of AI analyzers to assess HER2, ER, and PR status as an important characterization of breast cancer molecular subtypes improved the agreement of pathologists across IHC stains and thus molecular subtypes of breast cancer. Notably, this AI-assisted increase in concordance was more pronounced for low positive IHC cases with initial relatively low interpathologist agreement. The promise of AI-driven image analysis on precision oncology seen in this study will require further prospective investigation to validate possible real-world clinical impact.

### Supplementary Information

**Additional file 1**. Supplementary Method, Supplementary Results, Figures S1–6; Table S1–7.

## Data Availability

The datasets used and/or analyzed during the current study are available from the corresponding author for academic purposes upon request. The software was developed using Python programming language (version 3.9). The models are implemented using PyTorch v1.7.1 (available at https://github.com/pytorch/pytorch). The cell detection model and the tissue segmentation model are based on a proprietary implementation of DeepLabv3+ (open-source implementations available online, e.g., at https://github.com/VainF/DeepLabV3Plus-Pytorch), with a ResNet-34 and ResNet-101 backbone architectures (open-source implementation available at https://github.com/pytorch/vision/blob/master/torchvision/models/resnet.py). The data augmentation transformations and image manipulation routines are implemented using TorchVision v0.8.2 (https://github.com/pytorch/vision), Albumentations v1.2.1 (https://github.com/albumentations-team/albumentations), OpenCV Python v4.6.0.66 (https://github.com/opencv/opencv-python), and Scikit-image v0.19.3 (https://github.com/scikit-image/scikit-image). Mathematical and statistical operations are implemented using Numpy v1.23.1 (https://github.com/numpy/numpy), Pandas v1.4.3 (https://github.com/pandas-dev/pandas), Scipy v1.9.0 (https://github.com/scipy/scipy), and Scikit-learn v1.1.1 (https://github.com/scikit-learn/scikit-learn). Finally, the WSIs were read and manipulated using OpenSlide v3.4.1 (https://github.com/openslide/openslide) and the corresponding Python wrapper v1.2.0 (https://github.com/openslide/openslide-python). For any questions regarding the replication of results, the corresponding author can be contacted.

## Abbreviations

ADC: Antibody–drug conjugate
AI: Artificial intelligence
AJCC: American Joint Committee on Cancer
ASCO: The American Society of Clinical Oncology
BG: Background
CA: Cancer area
CAP: College of American Pathologists
CIS: Carcinoma in situ
DL: Deep learning
ER: Estrogen receptor
HER2: Human epidermal growth factor receptor 2
IHC: Immunohistochemistry
IoU: Intersection over union
ISH: In situ hybridization
OT: Other cell
P1: Pathologist 1
P2: Pathologist 2
P3: Pathologist 3
PD-L1: Programmed death ligand 1
PR: Progesterone receptor
ROI: Regions of interest
TNBC: Triple-negative breast cancer
WSI: Whole-slide image
