# Supplementary material for: Augmented interpretation of HER2, ER, and PR in breast cancer by artificial intelligence analyzer: enhancing interobserver agreement through a reader study of 201 cases
Source: Breast Cancer Res. 2024 Feb 23;26:31. doi: 10.1186/s13058-024-01784-y (PMC10885430; doi:10.1186/s13058-024-01784-y)
Supplement: Supplementary file 1 — Additional file 1. Supplementary Method, Supplementary Results, Figures S1–6; Table S1–7. [file 13058_2024_1784_MOESM1_ESM.docx]

## Supplementary Methods

*Detailed information of dataset for model development*

All tissues for human epidermal growth factor receptor 2 (HER2) artificial intelligence (AI) development were sourced from Cureline (Brisbane, CA, US), Aurora Diagnostics (Greensboro, NC, US), and Superbiochips (Seoul, Republic of Korea). All tissues were stained with Ventana anti-HER2/neu (4B5) (Ventana Medical Systems, Tucson, AZ, US), and were scanned by a P1000 scanner (3DHistech, Budapest, Hungary) with 40× magnification.

All tissues for estrogen receptor (ER)/progesterone receptor (PR) AI development were sourced from Cureline or Neogenomics Laboratories (Fort Myers, FL, US). The dataset consists of slides stained with Ventana ER SP1, Ventana PR 1E2 (Ventana Medical Systems), Dako ER EP1, and Dako PR PgR636 (Agilent Technologies Inc., Santa Clara, CA, US). Ventana ER/PR stained slides were scanned by P1000 scanner with 40× magnification, while Dako ER/PR stained slides were scanned by Aperio AT2 DX scanner (Leica Biosystems Imaging, Buffalo Grove, IL, US) with 40× magnification.

To augment the training dataset for ER/PR tissue model, an additional 270 programmed death ligand 1 (PD-L1) 22C3 (Agilent Technologies Inc.) immunohistochemistry (IHC)-stained dataset was added. The dataset complemented the relatively scarce Dako ER P1 and Dako PR PgR636 datasets in the ER/PR tissue dataset. Those tissues were sourced from Cureline and were stained with PD-L1 22C3 pharmDx IHC (Agilent Technologies Inc.). All PD-L1 IHC-stained slides were scanned by P1000 scanner with 40× magnification.

*Detailed information of annotation by board-certified pathologists*

The patches were annotated by many board-certified pathologists (115 pathologists for HER2, 105 for ER/PR). Pathologists annotated cells on the patches, 3+ (circumferential membrane staining that is complete and intense) / 2+ (weak to moderate complete membrane staining) / 1+ (incomplete membrane staining that is faint/barely perceptible) / 0 (no staining) tumor cells or other cells (OT) for HER2-stained images and 3+ (strong positive) / 2+ (intermediate positive) / 1+ (weak positive) / 0 (none) tumor cells or positive / negative OT for ER/PR-stained images [[1, 2]](https://www.zotero.org/google-docs/?E44MU9). They also segmented tissues on the patches as CA (cancer area; invasive breast cancer), CIS (carcinoma in situ), and BG (background; any tissue area that does not belong to CA or CIS) both for HER2 and ER/PR stained images. Numbers of annotated cells and areas of segmented tissues (mm^2^) in training, tuning, and internal test sets in the patch-level are summarized in Tables S2 and S3.

*Additional details of Cell Detection Model*

The cell detection task was approached as a dense segmentation challenge, utilizing the DeepLab v3+ segmentation model with a ResNet-34 for feature extraction [[3, 4]](https://www.zotero.org/google-docs/?64ec4S). It was trained to predict filled circles of 2.7μm diameter to match each marked cell location. The model predicts multiple dense maps; one signifies background areas (or non-cell regions) while the others represent the other cell types. The model's training incorporated patches sized 1024x1024 pixels. We processed the model's predictions (sized 1024x1024) to extract detected cells via a local peak finding algorithm [[5]](https://www.zotero.org/google-docs/?pKYofB). The training utilized the soft Dice loss function [[6]](https://www.zotero.org/google-docs/?V5h2tG).

*Additional details of Tissue Segmentation Model*

The tissue segmentation model employs a DeepLabv3, complemented with a ResNet-101 for feature extraction. It is designed to produce a pixel-wise probability map, which results in three dense maps corresponding to CA, CIS, and BG (or two maps for the ER/PR model). The models were trained using 1024x1024 pixel patches. Based on the model's predictions sized 1024x1024, the tissue classification for each pixel is derived by comparing probability values between CA, CIS, and BG channels for the corresponding pixel. The model was trained using the soft Dice loss function. For the ER/PR model, PD-L1 IHC-stained images were additionally used as a training set as mentioned above.

## Supplementary Results

*Performance of cell detection and tissue segmentation models - HER2*

The cell detection model achieved the best performance on OT (F1 score: 0.8208 [Tuning] and 0.8210 [Internal test]), while it showed the lowest performance on 2+ tumor cell (F1 score: 0.5654 [Tuning] and 0.4442 [Internal test]), as shown in Table S4. The performances were relatively lower in 2+ or 1+ tumor cell classes. In addition, when cells were misclassified into other classes, it was more common to be misclassified into similar cell types, such as misidentifying 1+ tumor cell as 2+ tumor cell (both being the same tumor cells) or misidentifying 0 tumor cell as other cell (both having the same HER2 negative expression status) (Table S5). The tissue segmentation model achieved better performance on CA (Intersection over Union [IoU]: 0.7581 [Tuning] and 0.7610 [Internal test]) than on CIS (IoU: 0.6098 [Tuning] and 0.4828 [Internal test]) (Table S4).

*Performance of cell detection and tissue segmentation models - ER/PR*

The cell detection model achieved the best performance on 3+ tumor cell (F1 score: 0.7966 [Tuning] and 0.7699 [Internal test]), while it showed the lowest performance on 1+ tumor cell (F1 score: 0.5921 [Tuning] and 0.5441 [Internal test]), as shown in Table S6. The performances were also relatively lower in 2+ or 1+ tumor cell classes. In addition, misclassification to similar cells was similar to the HER2 model. (Table S7). The tissue segmentation model achieved performances of CA comparable to the HER2 model (IoU: 0.7492 [Tuning] and 0.8017 [Internal test]) (Table S6).

## Supplementary References

[1. Wolff AC, Somerfield MR, Dowsett M, Hammond MEH, Hayes DF, McShane LM, et al. Human epidermal growth factor receptor 2 testing in breast cancer: American Society of Clinical Oncology–College of American Pathologists guideline update. Arch Pathol Lab Med. 2023;147:993](https://www.zotero.org/google-docs/?TFzUAq)–1000.

[2. Fitzgibbons PL, Dillon DA, Alsabeh R, Berman MA, Hayes DF, Hicks DG, et al. Template for reporting results of biomarker testing of specimens from patients with carcinoma of the breast. Arch Pathol Lab Med. 2014;138:595–601.](https://www.zotero.org/google-docs/?TFzUAq)

[3. Chen L-C, Zhu Y, Papandreou G, Schroff F, Adam H. Encoder-decoder with atrous separable convolution for semantic image segmentation. In: Proceedings of the European conference on computer vision (ECCV). 2018. p. 801–18.](https://www.zotero.org/google-docs/?TFzUAq)

[4. He K, Zhang X, Ren S, Sun J. Deep residual learning for image recognition. In: Proceedings of the IEEE conference on computer vision and pattern recognition. 2016. p. 770–8.](https://www.zotero.org/google-docs/?TFzUAq)

[5. Van der Walt S, Schönberger JL, Nunez-Iglesias J, Boulogne F, Warner JD, Yager N, et al. scikit-image: image processing in Python. PeerJ. 2014;2:e453.](https://www.zotero.org/google-docs/?TFzUAq)

[6. Milletari F, Navab N, Ahmadi S-A. V-net: Fully convolutional neural networks for volumetric medical image segmentation. In: 2016 fourth international conference on 3D vision (3DV). 2016. p. 565–71.](https://www.zotero.org/google-docs/?TFzUAq)

##
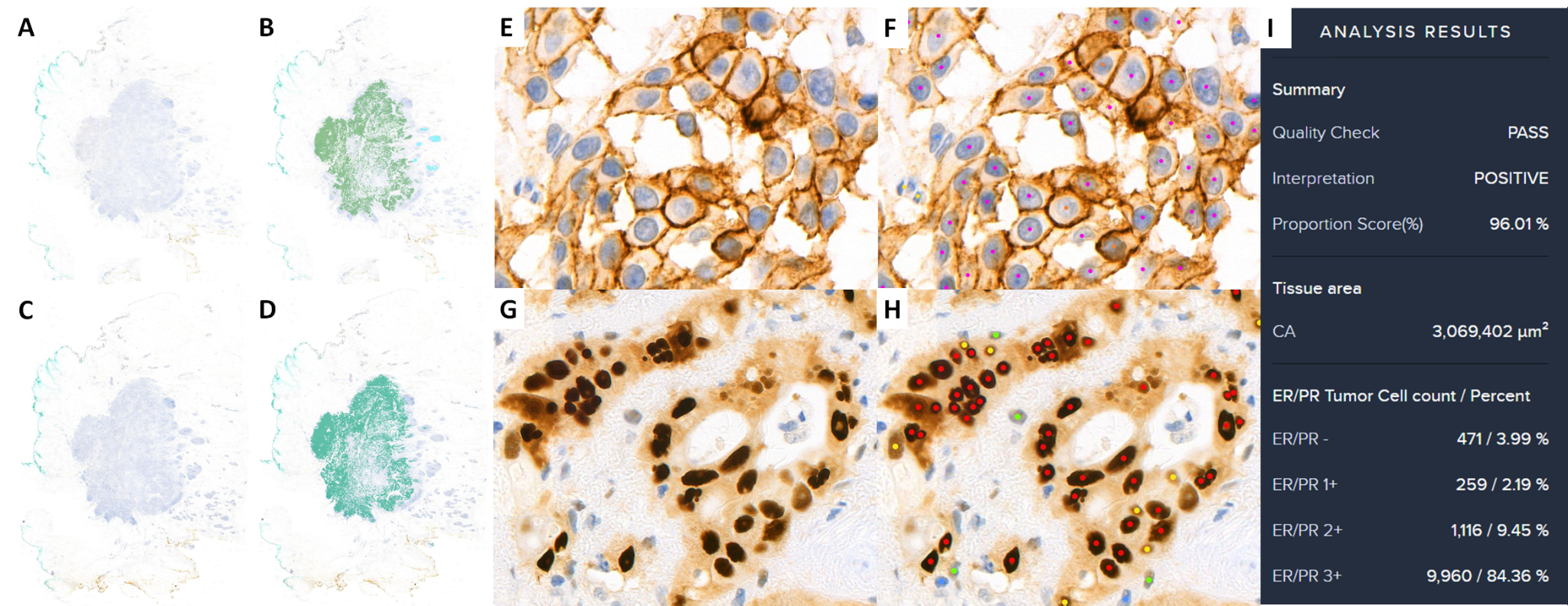


**Figure S1.** User interface of the whole slide image (WSI) visualizer displaying without and with AI analyzer results. (A) Before applying the tissue segmentation model for a HER2 (human epidermal growth factor receptor 2) stained slide. (B) After applying the tissue segmentation model for a HER2 stained slide. Dark green indicates cancer area and light blue indicates carcinoma in situ. (C) Before applying the tissue segmentation model for an ER (estrogen receptor) stained slide. (D) After applying the tissue segmentation model for an ER stained slide. Dark green indicates cancer area, and there is no carcinoma in situ class in the ER/PR (progesterone receptor) model. (E) Before applying the cell detection model for a HER2 stained slide. (F) After applying the cell detection model for a HER2 stained slide. Orange, pink, blue, and yellow color indicate 3+, 2+, 1+ tumor cells and other cells, respectively. (G) Before applying the cell detection model for an ER stained slide. (H) After applying the cell detection model for a ER stained slide. Red, yellow, and green color indicate 3+, 2+, and 1+ tumor cells, respectively. (I) Example of the results panel for an ER slide.


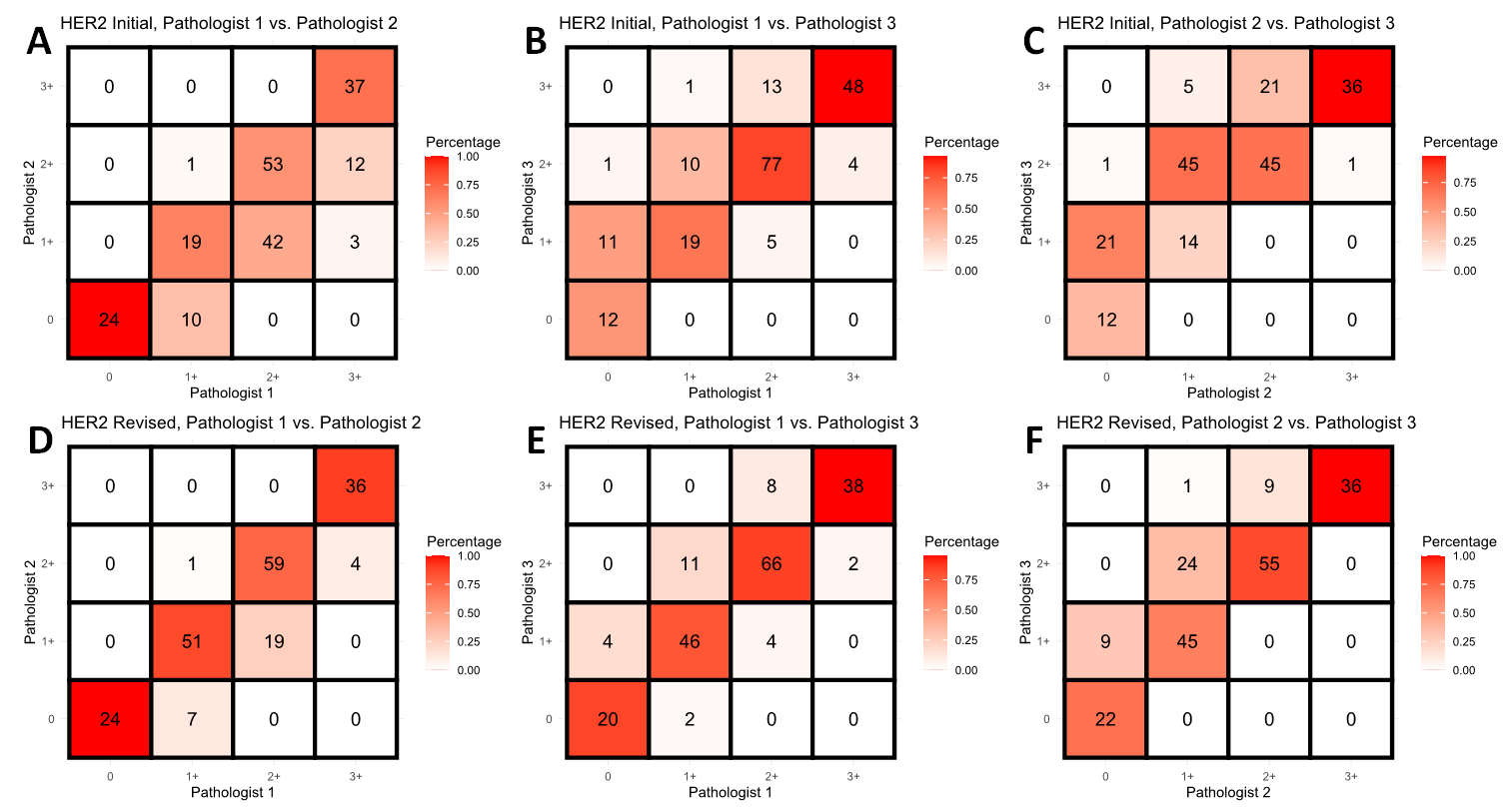


**Figure S2.** Confusion matrix for initial evaluation of HER2 (human epidermal growth factor receptor 2) between Pathologist 1 [P1] and Pathologist 2 [P2] (A), P1 and Pathologist 3 [P3] (B), and P2 and P3 (C). Confusion matrix for revised evaluation of HER2 between P1 and P2 (D), P1 and P3 (E), and P2 and P3 (F).


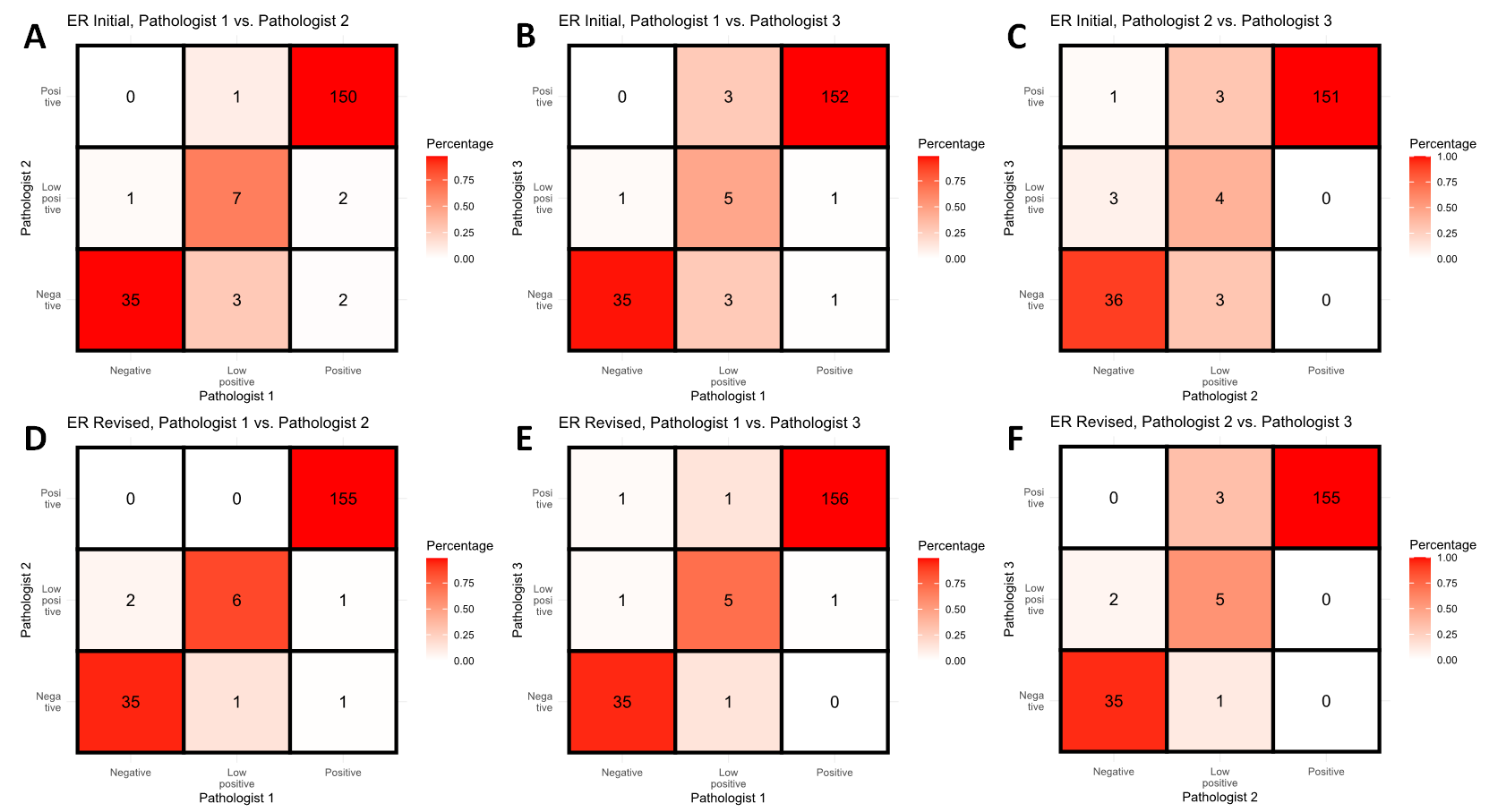


**Figure S3.** Confusion matrix for initial evaluation of ER (estrogen receptor) between Pathologist 1 [P1] and Pathologist 2 [P2] (A), P1 and Pathologist 3 [P3] (B), and P2 and P3 (C). Confusion matrix for revised evaluation of ER between P1 and P2 (D), P1 and P3 (E), and P2 and P3 (F).


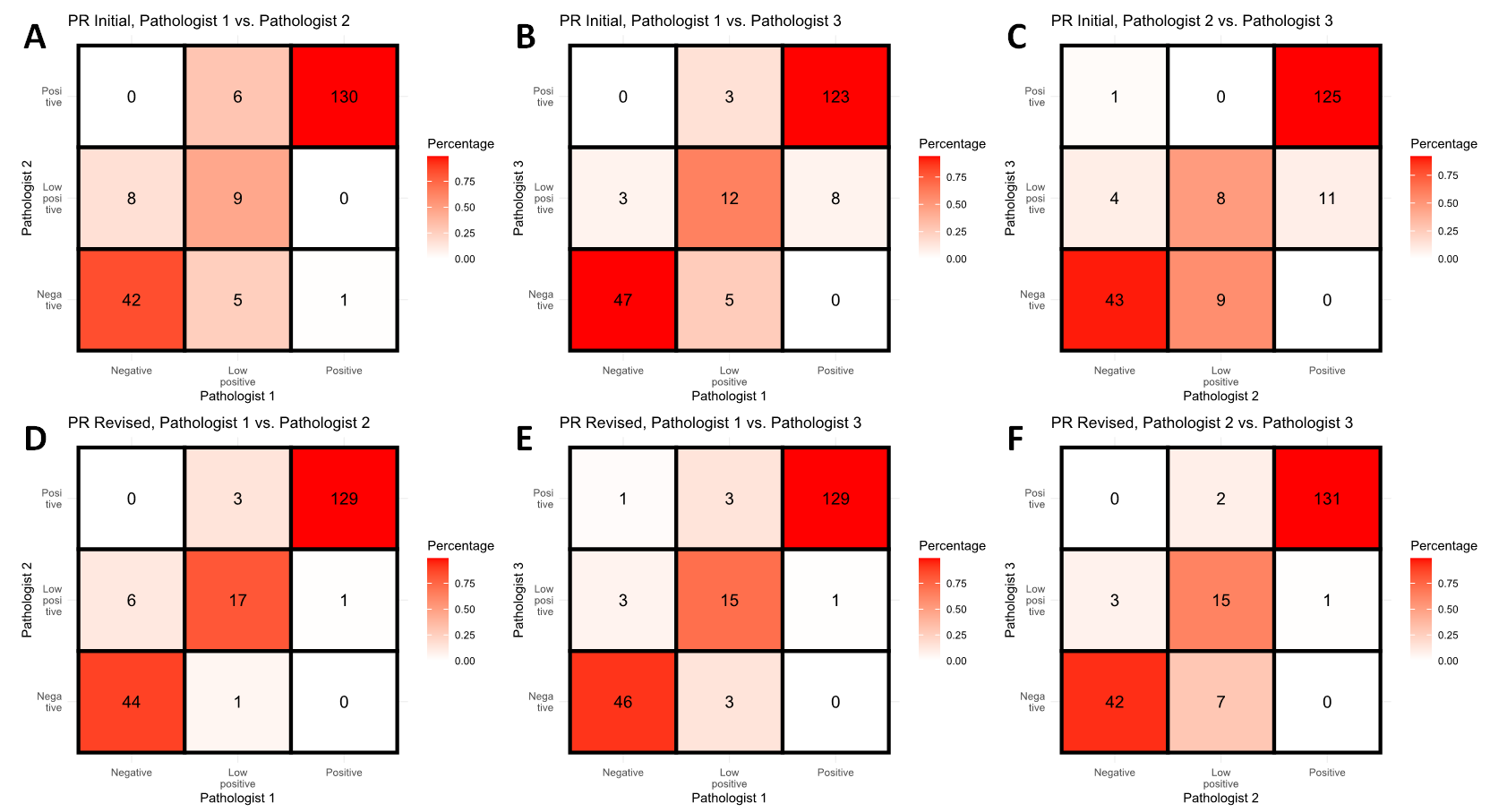


**Figure S4.** Confusion matrix for initial evaluation of PR (progesterone receptor) between Pathologist 1 [P1] and Pathologist 2 [P2] (A), P1 and Pathologist 3 [P3] (B), and P2 and P3 (C). Confusion matrix for revised evaluation of PR between P1 and P2 (D), P1 and P3 (E), and P2 and P3 (F).


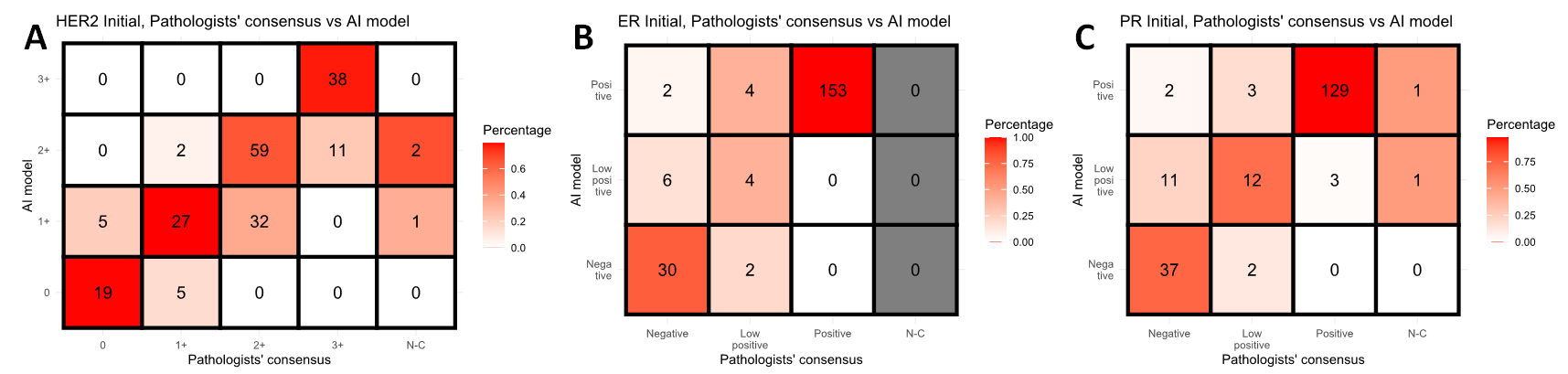


**Figure S5.** Confusion matrix for initial evaluation between the pathologists’ consensus and the artificial intelligence (AI) analyzer, in HER2 (human epidermal growth factor receptor 2) dataset (A), ER (estrogen receptor) dataset (B) and PR (progesterone receptor) dataset (C).


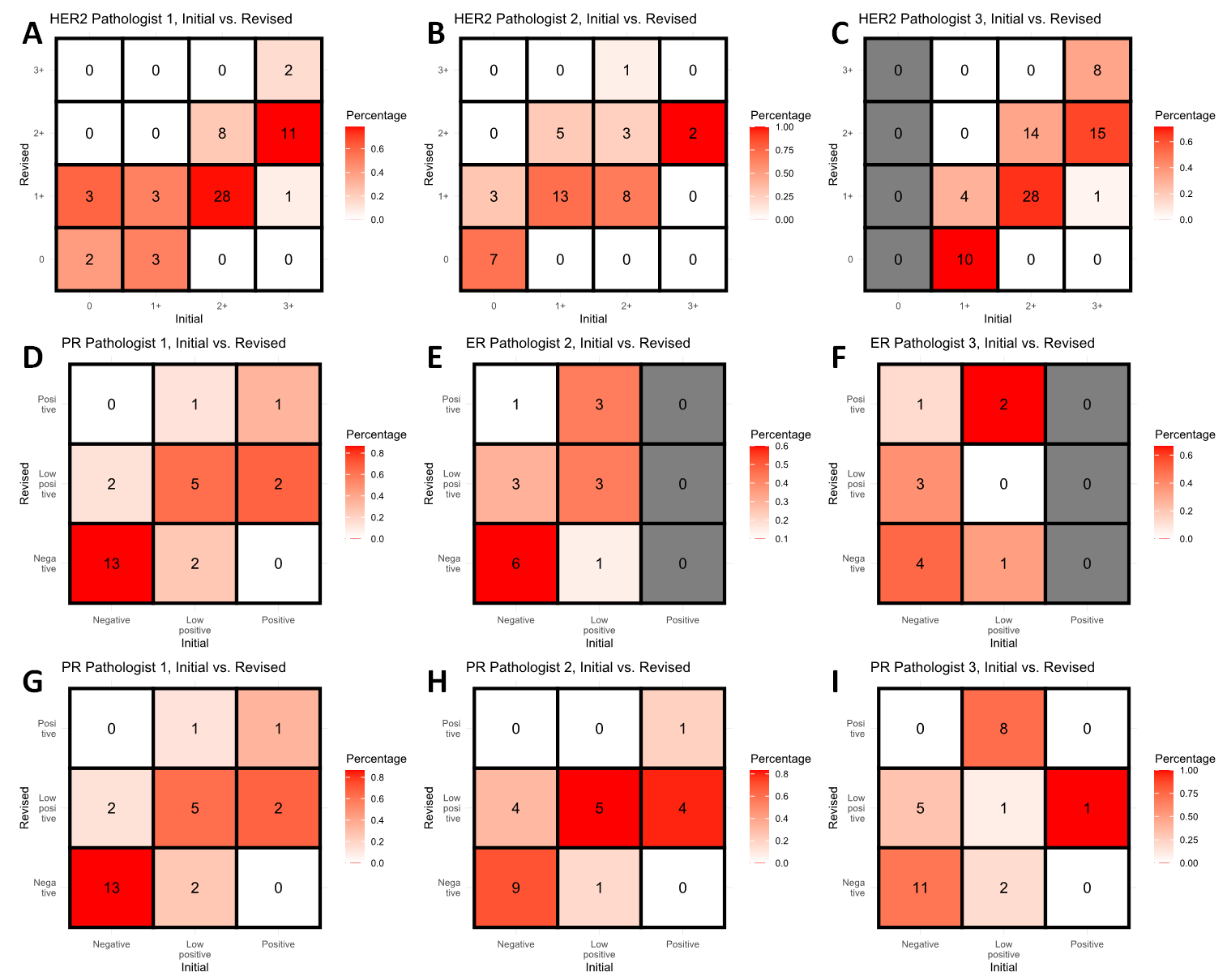


**Figure S6.** Confusion matrix for intra-rater changes (initial vs. revised) in revisited cases. (A-C) HER2 (human epidermal growth factor receptor 2) dataset. (D-E) ER (estrogen receptor) dataset. (G-I) PR (progesterone receptor) dataset.

**Table S1.** Numbers of whole slide images (WSIs) and patches assigned to training, tuning, and internal test set

|  | Staining | Training set | Tuning set | Internal test set | Total |
| --- | --- | --- | --- | --- | --- |
| WSIs | HER2 | 880 | 253 | 126 | 1,259 |
|  | ER (Dako/Ventana) | 487 (113/374) | 167 (59/108) | 90 (35/55) | 744 (207/537) |
|  | PR (Dako/Ventana) | 295 (97/198) | 120 (52/68) | 51 (28/23) | 466 (177/289) |
|  | PD-L1 22C3 | 270 | 0 | 0 | 270 |
| Patch (Cell) | HER2 | 5,614 | 1,679 | 856 | 8,149 |
|  | ER (Dako/Ventana) | 1,005 (486/519) | 320 (178/142) | 179 (100/79) | 1,504 (764/740) |
|  | PR (Dako/Ventana) | 780 (316/464) | 249 (133/116) | 123 (66/57) | 1,152 (515/637) |
| Patch (Tissue) | HER2 | 3,120 | 870 | 453 | 4,443 |
|  | ER (Dako/Ventana) | 2,648 (717/1,931) | 814 (224/590) | 406 (131/275) | 3,868 (1,072/2,796) |
|  | PR (Dako only) | 190 | 100 | 50 | 340 |
|  | PD-L1 22C3 | 2,691 | 0 | 0 | 2,691 |

ER: estrogen receptor, HER2: human epidermal growth factor receptor 2, PR: progesterone receptor

**Table S2.** Numbers of annotated cells and areas of segmented tissue (mm^2^) by pathologists in training, tuning, and internal test set of HER2(human epidermal growth factor receptor 2)

| Type | Class | Training set | Tuning set | Internal test set | Total |
| --- | --- | --- | --- | --- | --- |
| Cell (number) | 3+ tumor cell | 108,902 | 22,649 | 12,191 | 143,742 |
|  | 2+ tumor cell | 61,751 | 20,377 | 11,158 | 93,286 |
|  | 1+ tumor cell | 85,008 | 27,856 | 17,456 | 130,320 |
|  | 0 tumor cell | 306,679 | 97,889 | 41,216 | 445,784 |
|  | Other cell | 560,764 | 162,874 | 81,276 | 804,914 |
| Tissue (mm^2^) | Cancer area | 70,971.54 | 20,105.70 | 10,106.43 | 101,183.67 |
|  | Carcinoma in situ | 1,872.60 | 495.90 | 285.39 | 2,653.89 |
|  | Background | 239,255.86 | 66,398.40 | 34,899.12 | 340,553.38 |

3+ tumor cell: circumferential membrane staining that is complete and intense, 2+ tumor cell: weak to moderate complete membrane staining, 1+ tumor cell: incomplete membrane staining that is faint/barely perceptible, 0 tumor cell: no staining

**Table S3.** Numbers of annotated cells and areas of segmented tissue (mm^2^) by pathologists in training, tuning, and internal test set of ER(estrogen receptor)/PR(progesterone receptor)

| Type | Class | Training set | Tuning set | Internal test set | Total |
| --- | --- | --- | --- | --- | --- |
| Cell (number) | 3+ tumor cell | 50,725 | 5,804 | 5,967 | 62,496 |
|  | 2+ tumor cell | 45,035 | 7,588 | 7,077 | 59,700 |
|  | 1+ tumor cell | 41,420 | 5,576 | 4,762 | 51,758 |
|  | 0 tumor cell | 176,107 | 60,096 | 43,670 | 279,873 |
|  | Other cell, positive | 5,759 | 903 | 718 | 7,380 |
|  | Other cell, negative | 139,893 | 54,928 | 35,230 | 230,051 |
| Tissue | Cancer area (CA) | 194,383.23 (ER/PR only: 96,350.10) | 29,275.42 | 21,113.4 | 146,738.92 |
|  | Carcinoma in situ (CIS) | 18,803.58 (ER/PR only: 10,784.40) | 3,537.18 | 292.48 | 14,614.06 |
|  | Background (BG) | 339,740.10 (ER/PR only: 176,665.50) | 58,587.40 | 24,294.12 | 259,547.02 |

3+ tumor cell: strong positive, 2+ tumor cell: intermediate positive, 1+ tumor cell: weak positive, 0 tumor cell: none

**Table S4.** Performance of the cell detection and tissue segmentation of the HER2(human epidermal growth factor receptor 2) analyzer in tuning and internal test set.

| Type | Class | Tuning set | Internal test set |
| --- | --- | --- | --- |
| Cell (F1 score) | 3+ tumor cell | 0.7458 | 0.7010 |
|  | 2+ tumor cell | 0.5654 | 0.4442 |
|  | 1+ tumor cell | 0.5999 | 0.5637 |
|  | 0 tumor cell | 0.7842 | 0.7482 |
|  | Other cell | 0.8208 | 0.8210 |
| Tissue (Intersection over Union, IoU) | Cancer area | 0.7581 | 0.7610 |
|  | Carcinoma in situ | 0.6098 | 0.4828 |

3+ tumor cell: circumferential membrane staining that is complete and intense, 2+ tumor cell: weak to moderate complete membrane staining, 1+ tumor cell: incomplete membrane staining that is faint/barely perceptible, 0 tumor cell: no staining

**Table S5.** A confusion matrix of ground truth and predicted cell classes by the HER2(human epidermal growth factor receptor 2) analyzer

|  | Dataset | Prediction | | | | | | |
| --- | --- | --- | --- | --- | --- | --- | --- | --- |
| Ground truth | Tuning | Class | Background | 0 tumor cell | 1+ tumor cell | 2+ tumor cell | 3+ tumor cell | Other cell |
|  |  | Background | 0 | 6,231 | 2,760 | 2,142 | 2,529 | 17,581 |
|  |  | 0 tumor cell | 10,209 | 73,818 | 4,897 | 113 | 12 | 8,840 |
|  |  | 1+ tumor cell | 3,412 | 2,468 | 16,996 | 3,650 | 368 | 962 |
|  |  | 2+ tumor cell | 2,832 | 142 | 2,778 | 11,618 | 2,824 | 183 |
|  |  | 3+ tumor cell | 2,620 | 1 | 104 | 2,953 | 16,912 | 59 |
|  |  | Other cell | 20,737 | 7,903 | 1,376 | 303 | 80 | 132,475 |
|  | Internal test set | Background | 0 | 2,875 | 1,615 | 1,280 | 1,213 | 8,838 |
|  |  | 0 tumor cell | 4,125 | 29,285 | 3,048 | 162 | 34 | 4,562 |
|  |  | 1+ tumor cell | 2,059 | 1,244 | 10,149 | 3,220 | 222 | 562 |
|  |  | 2+ tumor cell | 1,475 | 20 | 2,516 | 5,208 | 1,821 | 118 |
|  |  | 3+ tumor cell | 1,395 | 0 | 164 | 2,220 | 8,363 | 49 |
|  |  | Other cell | 9,789 | 3,726 | 1,119 | 236 | 26 | 66,380 |

3+ tumor cell: circumferential membrane staining that is complete and intense, 2+ tumor cell: weak to moderate complete membrane staining, 1+ tumor cell: incomplete membrane staining that is faint/barely perceptible, 0 tumor cell: no staining

**Table S6.** Performance of the cell detection and tissue segmentation of the ER(estrogen receptor)/PR(progesterone receptor) analyzer in tuning and internal test set.

| Type | Class | Tuning set | Internal test set |
| --- | --- | --- | --- |
| Cell (F1 score) | 3+ tumor cell | 0.7966 | 0.7699 |
|  | 2+ tumor cell | 0.6865 | 0.6572 |
|  | 1+ tumor cell | 0.5921 | 0.5441 |
|  | 0 tumor cell | 0.7941 | 0.7655 |
| Tissue (Intersection over Union, IoU) | Cancer area | 0.7492 | 0.8017 |

3+ tumor cell: strong positive, 2+ tumor cell: intermediate positive, 1+ tumor cell: weak positive, 0 tumor cell: none

**Table S7.** A confusion matrix of ground truth and predicted cell classes by the ER(estrogen receptor)/PR(progesterone receptor) analyzer

|  | Dataset | Prediction | | | | | |
| --- | --- | --- | --- | --- | --- | --- | --- |
| Ground truth | Tuning | Class | Background | 0 tumor cell | 1+ tumor cell | 2+ tumor cell | 3+ tumor cell |
|  |  | Background | 0 | 16,215 | 1,194 | 692 | 448 |
|  |  | 0 tumor cell | 8,605 | 50,529 | 932 | 20 | 10 |
|  |  | 1+ tumor cell | 893 | 393 | 3,561 | 706 | 23 |
|  |  | 2+ tumor cell | 918 | 37 | 822 | 5,072 | 739 |
|  |  | 3+ tumor cell | 358 | 5 | 28 | 769 | 4,644 |
|  | Internal test set | Background | 0 | 12,961 | 781 | 954 | 413 |
|  |  | 0 tumor cell | 7,436 | 35,498 | 681 | 37 | 18 |
|  |  | 1+ tumor cell | 759 | 574 | 2,555 | 839 | 35 |
|  |  | 2+ tumor cell | 666 | 60 | 641 | 4,761 | 949 |
|  |  | 3+ tumor cell | 422 | 7 | 53 | 872 | 4,613 |

3+ tumor cell: strong positive, 2+ tumor cell: intermediate positive, 1+ tumor cell: weak positive, 0 tumor cell: none
